# Using qualitative research to explore intervention mechanisms: findings from the trial of the Learning Together whole-school health intervention

**DOI:** 10.1186/s13063-020-04688-2

**Published:** 2020-09-10

**Authors:** Emily Warren, G. J. Meledez-Torres, Russell Viner, Chris Bonell

**Affiliations:** 1 Faculty of Public Health and Policy, London School of Hygiene & Tropical Medicine, London, UK; 2 University of Exeter, Exeter, UK; 3 UCL Institute of Child Health, London, WC1N 1EH UK

**Keywords:** Randomised controlled trials, Realism, Whole-school interventions, Mechanisms, Restorative practice, Action groups

## Abstract

**Background:**

This study reports on qualitative research conducted within a randomised controlled trial to explore possible intervention mechanisms. It focuses on the ‘Learning Together’ whole-school intervention delivered in secondary schools in England from 2014 to 2017 aiming to prevent bullying and aggression and improve student health. Intervention schools received staff training in restorative practice, a social and emotional learning curriculum, and an external facilitator and manual to convene and run a student/staff action group tasked with coordinating the intervention, focusing this on local needs.

**Methods:**

Informed by realist approaches to evaluation, we analysed qualitative data to explore intervention mechanisms and how these might interact with school contexts to generate outcomes. Qualitative analysis drew on 45 interviews and 21 focus groups across three case-study schools and employed thematic content analysis to explore how intervention resources were taken up and used by local actors, how participants described the intervention mechanisms that then ensued, and how these might have generated beneficial outcomes.

**Results:**

The thematic content analysis identified three social mechanisms that recurred in participant accounts: (1) building student commitment to the school community, (2) building healthy relationships by modelling and teaching pro-social skills, and (3) de-escalating bullying and aggression and enabling re-integration within the school community.

**Conclusions:**

Our analysis provides in-depth exploration of possible mechanisms and the contextual contingencies associated with these, allowing refinement of the initial intervention theory of change.

**Trial registration:**

ISRCTN registry 10751359. Registered on 11 March 2014

## Introduction

This paper draws on qualitative data collected as part of the process evaluation within a randomised controlled trial (RCT) of the ‘Learning Together’ intervention to provide an in-depth description of participant accounts of the processes occurring, how these varied with local conditions in schools, and with what consequences. The paper then draws on this analysis to develop realist-informed hypotheses about how intervention mechanisms might interact with context to generate outcomes.

### Whole-school health interventions to prevent bullying and promote health

Bullying and aggression are common among secondary school students [1, 2] with important consequences for educational attainment and adolescent and adult physical and mental health [3–6]. Whole-school interventions are a promising approach to promoting student health across a range of outcomes, including bullying and aggression [7–10]. One approach within such interventions for which existing trials report evidence of effectiveness is for schools to convene an action group of students and staff who identify local actions that will encourage a more inclusive and engaging social and learning environment, with positive consequences for student health [11–13]. In some interventions, group decisions are informed by local data on students’ health needs and views about school [14, 15]. These have been found to be more effective if a member of the senior leadership team is on the action group and where students’ participation is taken seriously by staff and not seen as a tokenistic exercise [15].

A second promising approach within whole-school interventions is restorative practice, which involves responding to conflict not merely by punishing perpetrators but by understanding the causes of conflict, improving relationships, and re-integrating offenders back into the school community. This may take the form of a staff member leading a facilitated meeting between a bully and their victim, the victim being given the opportunity to describe the impact of the bullying, the bully being encouraged to acknowledge this harm and their responsibility for it, and the facilitator working with the two parties to enable healing in their relationship and the prevention of further problems. Although observational studies of restorative practice have generally been positive, before the current trial, there had been no experimental evaluations of this approach in schools [16–19].

A third promising approach is classroom interventions promoting social and emotional learning, for which there is strong evidence from RCTs that these promote student mental well-being, reduce conflict, and improve academic engagement and attainment, especially among students receiving free school meals and those underperforming in math and literacy, indicating a potential avenue for improving health equity [20–22]. Whole-school interventions are complex interventions involving multiple components that interact with each other and with context to generate emergent, socially contingent effects [23] theorised to be greater than had the components been introduced individually [24].

Drawing on this evidence, the Learning Together intervention was developed. Drawing on the work of Bernstein [25], the intervention was theorised to work by using action groups, restorative practice, and social and emotional learning to increase student commitment to the school's ‘instructional’ and ‘regulatory’ orders [26, 27]. The instructional order consists of processes of academic learning, and the regulatory order consists of school norms of behaviour and community [27]. Increasing student commitment to these orders occurs via a process of ‘reframing’: eroding ‘boundaries’ between and among students and staff, and between academic learning and broader student development. Existing theory suggests that, particularly among socio-economically disadvantaged students, such boundaries can persistently hinder student commitment to the instructional and regulatory school orders because relationships with teachers are insufficiently strong to engender a sense that school is something for them.

This can then contribute to students’ increased involvement in risk behaviours. This may be partly through students not being educated on how to avoid such behaviours [27] and partly through students committing to anti-school peer groups and risk behaviours, such as violence and substance use, which function as markers of belonging and status among such groups. The latter might occur particularly when ‘official’ markers of success in school seem unattainable to students who do not feel committed to school [28]. Eroding boundaries is theorised to promote commitment to the instructional and regulatory orders and therefore reduce bullying and aggression and promote student mental and physical well-being, but is something that requires a significant whole-school change in order to address these persistent structural influences on adverse outcomes.

Learning Together was evaluated through an RCT. The trial’s primary analyses indicated that the intervention was effective not only in reducing bullying victimisation but also in reducing smoking, drinking alcohol, using drugs, and in promoting mental well-being, psychological functioning, and health-related quality of life among students. Moderator analyses found effects were no greater for more socio-economically disadvantaged students but were greater for boys and for those reporting victimisation at baseline. There was also evidence that the intervention increased student commitment to school, providing some indirect evidence that the above theory of change might be plausible [29]. However, these trial analyses offer limited insights into the mechanisms of complex interventions and how these might play out across contexts to generate different outcomes in different schools. As suggested above, whole-school interventions can be thought of as contextually contingent with a consequent need to understand mechanisms and how these vary across the context of different students, schools, and school systems [30, 31].

### Randomised trials and realist evaluation

RCTs aim to estimate intervention effects while minimising confounding and other biases. However, RCTs have been criticised for being too narrowly focused on estimating the overall effects and failing to examine the mechanisms or how outcomes vary across contexts [32–34]. This view is supported by some reviews identifying but not explaining heterogeneity in effects among RCTs of similar interventions across different settings or populations [35, 36].

Realist evaluation aims to address this limitation by formulating and testing hypotheses about how contexts and mechanisms interact to generate outcomes, with such hypotheses being worded as context-mechanism-outcome (CMO) configurations [32]. Interventions are viewed as producing outcomes not directly but only via introducing resources into a setting which local actors may then use and in doing so may trigger ‘mechanisms’. Mechanisms are the consequences of people engaging with the resources of a programme or intervention in a certain context that bring about a change or effect. Mechanisms exist as tendencies which may or may not generate outcomes, contingent on ‘context’, or the conditions in which interventions are introduced. Context can influence whether intervention resources are taken up and used and thus trigger mechanisms (for example, because of social norms or structural constraints), as well as whether the mechanisms thus triggered will be sufficiently effective to generate ‘outcomes’ or instead be swamped by other mechanisms operating in that context. Outcomes represent the observable consequences of mechanisms [32]. Contexts, mechanisms, and outcomes are analytical categorises that social enquiry applies to make sense of the world. Realist evaluators generally assess CMO configurations using observational (non-randomised) comparisons of interventions deployed across and within differing contexts. A realist approach might therefore be helpful in moving beyond mere effect sizes to explore the contextually contingent mechanisms and impacts of whole-school interventions such as Learning Together. Within a realist-informed approach, qualitative research should be useful in exploring participants’ accounts of intervention mechanisms to assess whether these align with those theorised.

### Rationale for the current analysis

The present paper aims to draw on qualitative data collected within the trial’s process evaluation to explore participant accounts of intervention processes and how these might have played out with different consequences in different schools. The paper aims to develop a rich description of school conditions, intervention-related processes, and consequences from the perspective of the staff and students involved in implementing and receiving the intervention. Our ‘Discussion’ section then aims to reflect on these findings to consider their implications for the intervention’s underlying theory of change and to propose some CMO configurations in the light of our findings. Our qualitative research aimed to answer the following questions:

(i.) How did intervention participants describe the school context, the processes involved in participation, and the consequences of these?
(ii.) How did such accounts vary between schools, and what conditions relating to schools, staff, or students seem to explain these variations?

## Methods

### Trial design and methods

Trial methods are described in detail elsewhere [26, 29]. In summary, the intervention was evaluated from 2014 to 2017 using a superiority parallel-group cluster RCT randomly allocating (via computer-generated sequence) 40 state secondary schools (stratified by mixed/single-sex and rate of free school meal eligibility) in South East England to intervention or control (comprising usual practice) arms. Schools were recruited using email, phone calls, and a recruitment event targeting all eligible schools (excluding private schools, pupil referral units, schools exclusively for students with learning disabilities, and schools with ‘inadequate’ government inspection reports). Baseline surveys preceded allocation and consisted of self-completion questionnaires completed in privacy in classrooms by students nearing the end of year 7 (age 11–12) with similarly conducted follow-up surveys at 24 and 36 months. Head teachers consented to allocation and intervention. Surveys required students’ informed (written and oral) opt-in consent. Parents were informed and had the right to withdraw their children. The main trial analyses were intention-to-treat focused on primary and secondary outcomes at 36 months. Ethical approval for the trial was obtained through the Institute of Education Research Ethics Committee (18/11/13 ref. FCL 566) and the University College London Research Ethics Committee (30/1/14, Project ID: 5248/001).

### The Learning Together intervention

The intervention was delivered over 3 years and offered the following resources to schools: an intervention manual, a yearly report on student health needs and views about the school from annual student surveys, an external facilitator trained by the lead facilitator and research team to facilitate action group (for the first 2 years with the third year being facilitated internally by school staff), and a yearly social and emotional learning curriculum (dose 5–10 h of lessons per year). Restorative practice training was provided through a specialist organisation. In the first year, all staff received 2–3 h of training to understand restorative terminology and practices and integrate these into their everyday interactions with students. A further 3-day training was provided for 5–10 staff selected by schools to deliver restorative conferences convened to address instances of bullying, aggression, or wrong-doing and, depending on the seriousness of the incident, also involving parents or other external participants such as the police. The training involved interactive discussions, group work, and role plays.

Using these resources, school staff were encouraged to enact the following: form action groups comprising at least six students and six staff who meet six times per year to collaborate on and review the needs report, and formulate and implement local decisions to address the needs identified; oversee implementation of the social and emotional learning curriculum; and review school policies and rules to ensure that these support a restorative, engaging, and inclusive school environment. Schools were asked to recruit diverse students, including those less engaged with school, onto action groups and ensure their full participation. External facilitators were trained to ensure student contributions with the aim of ensuring local decision-making, and implementation was co-produced between staff and students [24]. Schools were also tasked with implementing restorative practice including preventative interventions in classrooms to avoid conflict and more intensive restorative conferences to address conflict. All delivery was face-to-face on the school site. Fidelity was generally good although more variable for the curriculum and in the third year [37].

### Qualitative data and analysis

The research took a qualitative approach to explore participant accounts of the way in which the intervention was viewed as being implemented and operating. Sampling was purposive in order to examine how accounts varied by factors thought likely to be important factors in the diversity of views expressed. The present analysis draws on qualitative data from three case study schools purposively sampled in the first year of the trial based on diversity regarding student free school meal eligibility, school type, facilitator, and facilitator-rated progress with implementation.

In each case study school, we aimed to conduct annual interviews with the staff member leading the intervention, the staff member leading the SEL curriculum, a student and staff member of the action group, and two student participants in restorative conversations. Three annual focus groups were also held: one with students on the action group, one with other students, and one with staff. Interviews were held in the first 2 years with the external facilitator. Telephone interviews with two other school staff plus one senior leader were held at the beginning of the intervention. Interview guides and prompts were tested during the intervention pilot. Interviews and focus groups were arranged in consultation with the intervention lead in each school, who were asked to select students diverse by engagement and students/staff diverse by involvement with the intervention. As with surveys, qualitative research proceeded on the basis of participants’ informed opt-in consent supplemented, in the case of students, with parental right of withdrawal. Except for phone interviews, all research occurred in private school rooms. Students were told that researchers wanted to know about their school generally, their opinions on bullying and aggression, and school-wide changes. All interviews were audio-recorded and transcribed in full. Interviews and focus groups lasted between 15 and 90 min. Field notes were also made after each data collection session (Table 1). Demographic information on participants was not collected.

**Table 1:** Data collection and response rate per school and year

Data source	Harper’s School				Meadowood School				St Anselm’s School				Totals by data collection type
	Y8	Y9	Y10	School total	Y8	Y9	Y10	School total	Y8	Y9	Y10	School total	
Action group interviews	–	2/2	0/2	2/4	–	4/2	0/2	4/4	–	2/2	2/2	4/4	10
Staff focus groups	1/1	2/1	0/1	3/3	1/1	1/1	0/1	2/3	1/1	0/1	1/1	2/3	7
Action group focus groups	1/1	1/1	0/1	2/3	1/1	1/1	1/1	3/3	1/1	0/1	1/1	2/3	7
General student focus groups	1/1	1/1	0/1	2/3	1/1	1/1	1/1	3/3	1/1	0/1	1/1	2/3	7
Student participant in restorative practice interviews	1/2	3/2	0/2	4/6	2/2	2/2	2/2	6/6	2/2	3/2	2/2	5/6	15
Curriculum coordinator interviews	0/1	0/1	0/1	0/3	0/1	1/1	1/1	2/3	0/1	1/1	0/1	1/3	3
Staff Interviews	3/3	–	0/1	3/4	3/3	–	1/1	4/4	3/3	–	1/1	4/4	11
Facilitator interviews	1/1	1/1	–	2/2	1/1	1/1	–	2/2	1/1	1/1	–	2/2	6
School totals				18				26				22	
Number of interviews and focus groups completed													66

The analysis involved thematic content analysis. The analysis sought to identify themes pertinent to our research questions inductively from the data. Data were organised in Nvivo QSR. Coding occurred transcript by transcript ordered chronologically, school by school, and in waves, with initial open codes identifying recurring themes in participant accounts, following by further axial coding which drew on open codes to develop a deeper analysis of the social phenomena under investigation: participant accounts of mechanisms and how these interacted with context to generate outcomes.

Although we used thematic content analysis, identification and interpretation of themes were nonetheless sensitised by ideas derived from a variant of a grounded theory known as dimensional analysis. The dimensional analysis aims to draw on qualitative data to delineate social phenomena in terms of their context (the boundaries of a phenomenon), conditions (factors that facilitate, block, or shape phenomena), process (actions or interactions involved in phenomena), and consequences (effects or outcomes of phenomena) [38, 39]. Although the terminology used within dimensional analysis differs from that of context, mechanism, and outcome used within realist evaluation, we felt that this framework of context, conditions, processes, and consequences provided by dimensional analysis could be used as a heuristic device within our thematic content analysis, allowing us to dissect participant accounts of the mechanisms triggered by their engagement with Learning Together resources and how these were described as interacting with school context to generate outcomes. With this in mind, axial coding was built on open coding to, where possible, identify and group together open codes describing the context, conditions, processes, or outcomes of mechanisms as they were described in participant accounts. Our results are thus presented in terms of participant accounts of conditions, processes, and consequences. In the ‘Discussion’ section, we reflect on these findings to consider their implications using the realist language of context, mechanisms, and outcomes.

## Results

Thematic content analysis identified a central theme of the intervention resources being used by staff and students to build an inclusive and cohesive school environment. This overarching theme in turn comprised three themes describing three mechanisms: (1) improving student commitment to the school community, (2) building healthy relationships by modelling and teaching pro-social skills, and (3) de-escalating bullying and aggression and enabling re-integration within the community. All three of these themes were apparent in participants’ accounts as processes which had the potential to reduce conflict in schools and each comprised smaller processes. These themes, and their constituent sub-themes describing different aspects of these processes, are described below, in each case with a description of the conditions within which processes were reported to arise and their consequences. Table 2 depicts these conditions, processes, and consequences in more detail. First, however, we present a brief description of the overall context of our case study schools, which define the boundaries of our empirical research. Pseudonyms are used throughout. We indicate student year groups 8–10 which involve students age 11/12 through to 13/14 years.

**Table 2:**
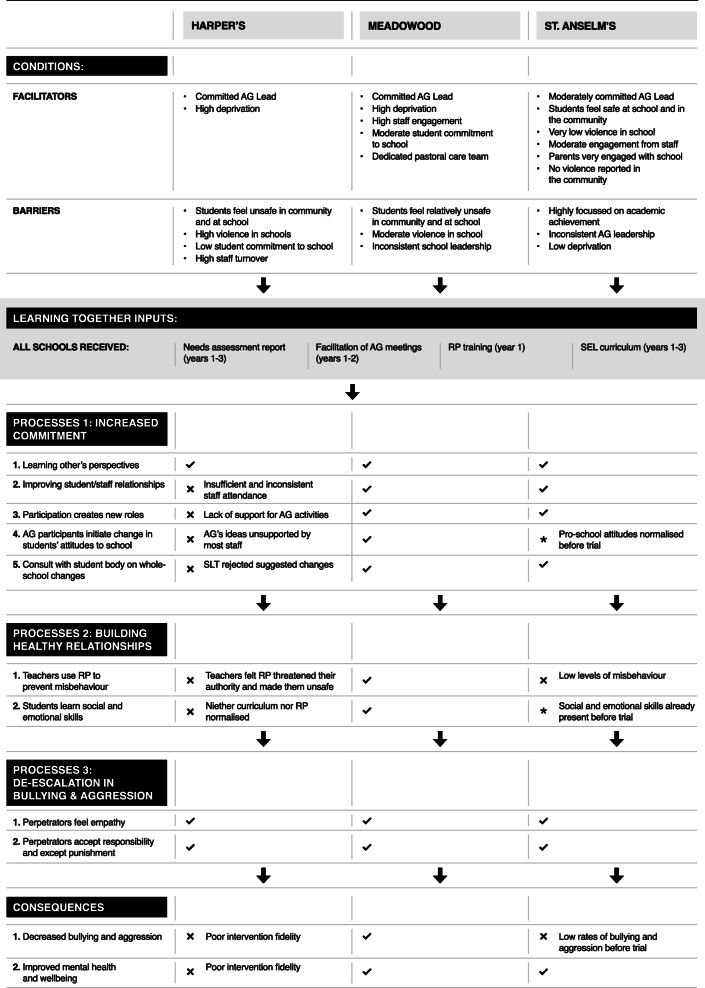
Conditions, processes, and consequences of Learning Together in case study schools

### School contexts

#### Harper’s School

Harper’s School is located in a deprived area of inner London, with a high rate of free school meal eligibility and of English as an additional language among students. Teachers described Harper’s as a school in which students often felt compelled to ‘act tough’ both in school and the surrounding community. Students described violence as being common. When asked how he felt after seeing one student break a boy’s nose and a boy threatening a girl with a knife, the student responded:

I just thought...this is school. It’s not supposed to happen, but it does. (Student, Harper’s, RP interview, year 9)

Staff reported feeling overwhelmed and under-supported, and the school had volunteered for the trial in order to address this. While some teachers had warm relationships with students, others were described as violent. Students reported that good teachers were leaving the school:

[Teachers] that really cared, they already left the school. Because, like, the school is, like, getting worse. The teachers... the good teachers are now, like, ...they’re all going. So, the bad ones, they’re now staying. (Student, Harper’s, RP interview, year 9)

Both students and staff commented that ‘good’ students did not want to be involved in anything beyond the minimum requirements. When asked why, a staff member reported:

Because the good students want to be invisible … They don’t want to be thought of as being geeks or being part of the establishment. They just want to get through school, get their qualifications and move on. (Staff, Harper’s, interview)

#### Meadowood

Meadowood is located in outer London, with more than 50% of students eligible for free school meals and with English as an additional language. Meadowood had recently transitioned through several head teachers. The school had a large body of students disengaged from education and involved in anti-social behaviour, whom one staff member described as having parents who give their children ‘a very long rein at home’. Some teachers reported their struggles to enforce boundaries:

They come here and when someone says “No, actually, you know, it’s no.” And that’s quite a new concept for a lot of them. (Staff, Meadowood, staff FGD)

Some students and teachers reported feeling unsafe at times. Students reported needing, or at least seeming, to be aggressive to get by:

When we’re in school, it’s like we do have this shield where we just try and protect ourselves. And teachers don’t really understand that: why we get angry or why we do this. (Student, Meadowood, RP interview, year 8)

Many staff members reported feeling safer when they could assert their authority over students, and hence, some were hesitant to use the more inclusive language of restorative practice. However, the school maintained a strong pastoral care team to support students with complex needs, and this group had been instrumental in the school volunteering for the trial. Despite its problems with some aggressive students, the external facilitator described Meadowood as ‘quite nice, a happy place to be’ and students described receiving ‘a brilliant education’ from teachers that cared not only about their academic progression but also about who they are and how they feel.

#### St. Anselm’s

St. Anselm’s was located in an affluent area outside London with lower rates of free school meals eligibility and English as an additional language. One staff member commented:

The vast majority of students that come to us are very well off … . And, typically, each year I would say we get about a thousand people apply for two hundred spaces....They want to come here. We’re not a second-choice school. (Staff, St. Anselm’s, SLT interview)

No participants reported aggression as common in St. Anselm’s, and when students did behave anti-socially, the school was able to arrange tailored support. Students reported positive relationships with staff and feeling cared for and supported. Parents, the school, and the students themselves all expected academic excellence.

### Increasing student commitment to the school community

This theme describes a process of increasing student commitment to the school community. It was composed of a number of sub-themes describing different parts of this process.

One sub-theme described a process involving the creation of new roles whereby staff and students on the action group came to share views and experiences. This could generate consequences of increased empathy and collegiality between staff and students, and consequently improved student relationships with staff and a desire to avoid anti-school behaviours.

Action groups functioned as a safe space within which students and staff could share their experiences and views and listen to those of others. As one staff member described:

I think that the students will certainly enjoy the fact that we’re doing something like this so they can be involved in it and that they can actually have their voice heard, that they can feel safe at school, that they can feel engaged with the teachers, that they can feel they’re listened to. (Staff, Harper’s, staff interview)

Interviews with students suggested that they valued the conversations that occurred in action group meetings and the insights provided into other members’ views and feelings. For example, when asked what was the best thing about being on the group was, one student replied:

I think mainly just having other people’s, seeing other people’s views and seeing how... if we had the same views or... hearing someone else’s point of view and thinking, “Oh yeah.” (Student, Meadowood, AG interview, year 9)

Action groups provided an opportunity for staff to enact new roles discussing the challenges that they faced. In Meadowood’s group, for example, staff spoke about how just one misbehaving student could disrupt an entire lesson. By being exposed to teachers’ reports of the impact of student misbehaviour in a non-confrontational setting, students began to see their teachers as people with feelings and not merely authority figures. As one teacher put it:

I suppose in some respects the students need to know that, ultimately, you’re a human being. Because I think they forget that we’re a human being and we have feelings. (Staff, Meadowood, staff FGD)

Another sub-theme described how the process of students working with teachers on the action group helped build relationships between students and staff by working collectively in co-producing decisions. This was especially important as some students on the action group were selected because of their perceived disengagement with school or previous experience with bullying. The relationship between students and staff was said by students in one focus group to feel:

much more respectful … yeah, they treat you with the same amount of respect as they would do their colleagues (Students, St. Anselm’s, AG student FGD, year 10)

Students reported that the sense of collegiality developed through collaborative processes of co-production in action group meetings had the consequence of giving them a more personal view of the teachers and motivating them to work harder for the staff they had learned to respect:

If you have a bond with your teacher... you want to do well for the teacher because you feel like she’s paid attention to you and gave her respect. And the way you can respect her back is by working hard. (Student, St. Anselm’s, AG student FGD, year 8)

In terms of the conditions required for such processes, this appeared to be contingent on a critical mass of staff and students participating in the group and the group being well-facilitated, such as in Meadowood and St. Anselm’s. In contrast, only one staff member regularly attended meetings in Harper’s, therefore limiting students’ opportunities to understand staff perspectives.

A further theme was student participation in the action group enabled them to enact new roles of contributing to the school community. This process could generate consequences of students experiencing a sense of agency and belonging at school but was contingent on the extent to which action groups were well attended and effective bodies [40]. For example, students participating in St Anselm’s action group were asked by the staff to help explain findings in the student needs report. Thus, students were accorded the role of an expert in contrast to the more conventional role of novice learner. Furthermore, all students on the action group possessed pertinent knowledge from their own experiences of school and from friends’ experiences. This was in contrast to the normal student role, for which many less-academic students felt they lacked the knowledge to render this performable. As one staff member described:

[Analysing the needs report] has then really highlighted to us as staff in school where we need to be focusing some particular work with the students... And then we’ve obviously taken a lot of advice and input from the students as to how they would like things to change in the school. So … students [are] feeling like they’re actually having an input. (Staff, St Anselm’s, staff interview)

Building relationships and being given a concrete task were especially important in Meadowood and Harper’s where students generally did not feel that they were listened to. In these schools, where students reported feeling intimidated at the beginning of the intervention, staff described how student confidence could grow:

What I’ve tried to do is tried to be able to break away into smaller groups as often as we can in those meetings so that they can be able to have a one-on-one with the members of staff. And as soon as that happens … that’s when really the conversations start in there. (Staff, Meadowood, AG interview)

Participation in Meadowood’s action group was seized on by some students as an opportunity to transform their experience of school:

Interviewer: Were you happy to be part of the action group?

Student: Yeah. When Miss Baker told me about it and I was... sad that I had to miss some of my favourite lessons. But I wanted to because I was getting bullied as well and I wanted to be able to stop it. (Student, Harper’s, AG student FGD, year 8)

The above processes would have had only limited consequences if they were restricted to students on the action group. However, further analysis revealed several processes via which the action group appeared to have consequences for other students. Many students who sat on action groups had previously been involved in anti-school peer groups and could share their experiences and thus initiate broader changes in student attitudes:

We have a boy … A proper naughty boy. But he has shown such maturity in the final part of his year-11 and he’s been, I think, an outstanding student in year-12. But all the kids know who he is or they know of the local family. And so, if he’s on board [with the action group], that sends a really important message. And I think that is critical. (Staff, Meadowood, staff FGD)

Another sub-theme described how the action group could have broader consequences via consulting with other students and/or generating actions which affected other students. For example, in St. Anselm’s, teachers reported that students on the action group developed new rules for student behaviour in a remodelled common room. Students consulted with their peers and developed new rules. Staff reported that, because a broad group of students had contributed to the process, students felt consulted and were more likely to respect the rules.

In terms of the conditions required for such processes, these appeared to be contingent on meetings being well-attended, well-led, and achieving results. In Meadowood, student participation was encouraged by the pastoral care team sitting on the action group as well as the commitment of the senior staff member leading the group. Similarly, in St. Anselm’s, the staff member leading the action group encouraged students to contribute ideas and ensured that their suggestions were implemented. Students’ sense of agency and contribution was less apparent in the narratives of students in Harper’s. While the only staff member consistently attending the Harper’s action group meetings tried to nurture her students, the lack of support from other staff and the dislocation of the action group from broader school structures made this challenging. In schools like Harper’s, where action groups were less effective in achieving action, students’ efforts to advocate for change could result in disappointment.

### Building healthy relationships by modelling and teaching pro-social skills

The second key theme described a process of building healthy relationships by modelling and teaching pro-social skills. This theme could be understood in terms of a number of sub-themes, each describing smaller, more subtle processes.

One such sub-theme was that when teachers were empowered and able to use a restorative practice to prevent misbehaviour in classrooms, students appeared to develop increased empathy, more respectful relationships, and reduced conflict. Meadowood staff integrated restorative practice into their normal classroom management practice to prevent and respond to consistent, low-level disruption and improve student/staff relationships. When a student was acting inappropriately in class, teachers would try to resolve the issue using restorative language. If the student did not change their behaviour, they were sent out of the room. Then, either the teacher or a member of the school’s pastoral team would meet with the student to have a restorative meeting, aiming to explore why they were misbehaving followed by a brief re-introduction meeting. According to staff, this process reduced frustration and animosity between students and teachers:

Because we do have a great number of students who are children in need. We have a lot of students who obviously have issues outside of school and they bring those issues within school. We wanted to be able to implement that restorative nature in the work that every member of staff was doing with kids within the classroom. (Staff, Meadowood, AG interviews)

Like action groups, such processes engendered a sharing of perspectives and increased empathy between students and staff. One teacher commented:

There’s a very easy way [to talk to challenging students] in the sense of being respectful and … considering what those students’ feelings are all the way through that because they are humans too, you know....That, you know, when members of staff get to that point where they actually look over that and they kind of go, “OK, well if I talk to you and I say to you, you know this isn’t what I expect in my lesson. I am giving you a half-an-hour detention, but in that half-an-hour detention you and I can be able to speak about what the problems are.” (Staff, Meadowood, AG interview)

In terms of necessary conditions, this sub-process was contingent on conditions of a critical mass of classroom teachers agreeing that student behaviour was a problem and that preventative restorative practice was a plausible response. Meadowood had high levels of classroom disruption and a pastoral care team who led the implementation of this approach and was able to support teachers. Sufficient numbers of teachers implemented the new practice informed by a recognition that previous approaches had not worked:

There were those moments where I would scream and shout at kids you know. And have a go at them and try to be able to make them see my way in a forceful way. It has no impact. (Staff, Meadowood, AG interview)

There was less evidence of such processes in the other schools. Data from Harper’s do not indicate sufficient engagement from staff to normalise the restorative practice. In St. Anselm’s, the limited need for restorative practice diminished staff’s need to use it consistently.

Another sub-theme described a sub-process of students learning social and emotional skills, which appeared to generate consequences of students being better able to avoid or resolve conflict. Students at Meadowood who had previously been involved in bullying and aggression described how they learned to manage emotions and social relationships constructively:

I like the fact that we get...that someone’s actually teaching us how to control our emotions, so if there’s an argument we know how to stop it … Instead of kicking off at your friends, just talk with a normal tone and just apologise and see how it goes from there. (Student, Meadowood, AG student FGD, year 9)

Students learned new tactics such as asking someone to stop doing something upsetting or pausing between feeling angry and responding. This process appeared to be contingent on teachers delivering the curriculum with fidelity and students having unmet needs in this area, as was the case in Meadowood. In St Anselm’s, staff reported that students already had well-developed coping skills. In Harper’s, only one unit of the curriculum was taught.

### De-escalation of bullying and aggression among a core group of students

The third key theme identified in our analysis describes a process of de-escalating bullying and aggression among a core group of students heavily involved in such activities. One sub-theme described a process whereby perpetrators of bullying or aggression began to feel empathy for victims through restorative practice conferences.

In Harper’s, one student had encouraged his friend to take a photograph of a boy on the toilet. The two then shared the image via social media. At first, the student was unrepentant:

[During the meeting,] I was like, “I didn’t really do anything” … Then they start staring at me; I’m like, “Don’t look at me, I didn’t do anything.”

However, seeing how devastated the photographed boy was made the perpetrator feel empathy, shame, and contrition:

And when we came in, it’s just, like... at first I was laughing, because I just felt it was hilarious for him... someone to be taking the pictures of him in the toilet. But then when I just saw him there sitting down at this table and his eyes were all red from the tears... I just don’t... it just came to me and just shocked me. That that could have happened to me really, it wouldn’t be nice.

A second sub-theme was that such processes could generate perpetrators’ recognition of their responsibility and acceptance of punishment. The same boy who encouraged the photo taken in the toilet reported that:

I normally would have been moaning [about being punished], saying “No” … But this time I actually felt what I had done was really wrong. It just makes me realise... I mean it’s ... just when I saw him sitting there in that state. (Student, Harper’s, RP interview, year 8)

Restorative conversations appeared to be more consequential when they removed bullies from their peers and were forced to speak directly with the person who had been hurt by their behaviour.

[I would have wanted a one-on-one meeting] Because I think maybe because all of us were in one room – you know, reputation, you don’t want to look, you know, smaller than one person and look you know weaker or more emotional than the others who were involved in the kind of like, oh I don’t really care. So I think maybe having that...you know, one-on-one rather than having everybody together... (Student, Meadowood, RP interview, year 9)

Contrasting the three schools, the data suggest that for restorative practice to be widely and effectively enacted was contingent on conditions of broad support from staff as well as significant levels of bullying or aggression. The conditions for Learning Together’s implementation at Harper’s were characterised by students in terms of substantial problems with bullying and aggression (so that the processes triggered by restorative practice might have been effective) but low staff engagement with the intervention (so restorative practices were rarely used). At Meadowood, the reported conditions also involved high rates of aggression and misbehaviour but also included a pastoral support team committed to the intervention so that restorative conversations were routinely used. The conditions at St. Anselm’s included staff committed to restorative practice but also low rates of conflict so that the processes triggered by its use were unlikely to produce significant consequences in terms of reduced bullying or aggression.

## Discussion

### Summary of key findings

This study aimed to develop a rich description of participant accounts of, drawing on concepts from dimensional analysis, the processes involved in a whole-school health intervention in secondary schools in South East England and how these might interact with local conditions to generate consequences, drawing on qualitative data from a process evaluation embedded within the RCT. Within a thematic content analysis, the concepts of conditions, processes, and consequences were used heuristically to interpret qualitative data.

In terms of our first research question, regarding how participants described the school context, the processes involved in their participation with the intervention, and the consequences of these, our analysis suggested several possible social processes involved in the intervention. These involved interactions between participants’ agency, the school’s structural context, and the possibilities introduced by the intervention’s resources [32, 41]. Firstly, participants described a process by which the intervention helped the student to develop a commitment to the school community via staff and students on the action group coming to share views and experiences through collaborative co-production activities [24], relationships improving, students participating on the action group empowering them to enact new roles of contributing to the school community, and influential students on the action group bringing about changes to broader student attitudes. The second process involved building healthy relationships by modelling and teaching pro-social skills. This involved teachers using a restorative practice to prevent misbehaviour in classrooms and students learning social and emotional skills. The third process involved de-escalating bullying and aggression and enabling reintegration by giving offenders the opportunity to learn empathy and take responsibility for their actions. All three processes were presented as helping to create more inclusive and cohesive school environments. It was rare that a single process was described by itself as being sufficient for creating change. Participants described inter-related chains of processes that could be assembled and work together to reduce bullying and aggression. For example, students sharing their experiences with staff would likely be insufficient to decrease bullying, but some schools may be able to reduce bullying by improving commitment to the school community.

Action groups and restorative practice were novel firstly in terms of privileging personal perspectives, which meant that students could draw on their experiences and views, and knowledge about their friends’ experiences and views, i.e. their cultural and social capital [42], to contribute. Secondly, action groups and restorative practice sessions involved a small number of individuals. This meant that both student and staff performances felt less precarious than most classroom interactions. Thus, a new role, that of a community member, was created which both students and staff could enact, transcending their previously separate (and often oppositional) roles. In enacting this role, students and staff had more opportunities to see each other’s perspectives and feel empathy for and collegiality with each other. This new role also created opportunities for new forms of pro-school identity and status, especially for students who had previously felt disengaged from school. In inhabiting this new role, students reported feeling more confident in working with adults and empowered to contribute to discussions and decisions. Such processes could affect a considerable number of students in schools, for example, when the restorative practice was used widely within classrooms to prevent conflict or when action group activities cascaded out to affect a broader group of students, such as where a broader group of students were involved in re-writing of school rules.

In terms of our second question, regarding how accounts varied between schools and what conditions relating to schools, staff, or students seemed to explain these variations, our analysis described numerous ways in which participant accounts varied between schools and what conditions relating to schools, staff, or students seemed to explain these variations. For example, the process of developing empathy between staff and students on action groups depended on consistent participation on actions groups and these being well run. Students’ growing confidence depended on groups being valued and achieving results. For bullying to be effectively addressed, the school needed to identify it as an issue and gain commitment from staff to use restorative practice widely to address it. In schools where a critical mass of students regularly displayed aggressive behaviour, schools benefitted from implementing the social and emotional learning curriculum with fidelity and/or using restorative practice widely as a preventative approach, which in turn required the involvement of senior staff who supported the intervention, as the divergent experiences at Harper’s and Meadowood show.

### Limitations

The analysis presented here draws on data from only three schools but also, within these, from multiple interviews and focus groups conducted over 3 years. Sampling relied on the school intervention lead selecting interview participants. Although the evaluation team did ask to speak with students from a broad range of experiences and levels of school engagement, it is possible that staff chose students who might reflect more positively on the school. However, given how freely some students expressed negative feelings towards the schools, this concern is not overwhelming.

Although there were some data on how intervention-related processes might have reduced bullying and aggression, there were fewer data on how increasing student commitment to school might have reduced other risk behaviours, such as smoking, or promoted mental or physical health. This gap was partly because of the process evaluation’s inevitable focus on intervention delivery and participation and the more proximal impacts. It might also have arisen because participants had less insight into more distal impacts. Nonetheless, there is broader evidence that increasing student commitment to school can reduce risk behaviours such as smoking and drug use and promote mental and physical health [30, 43]. For example, previous qualitative research describes mechanisms whereby student commitment to the school is associated with disengagement from risk behaviours. This appears to occur via commitment to school providing a sense of identity and status, so that young people feel less need to engage in behaviours such as smoking and drug use as alternatives source of identity and status within anti-school peer groups [28]. Furthermore, there is quantitative evidence from the trial that students did experience both increased commitment to school and reductions in smoking, drinking, and drug use as well as improvements in mental well-being and psychological functioning [29].

### Implications for research and policy

First, let us consider the implications of our results in terms of the intervention’s original theory of change before moving on to propose realist-informed CMO configurations in line with our findings.

Our results are broadly in line with the intervention’s original theory of change in terms of the intervention plausibly eroding boundaries between staff and students [27]. Our findings provide extra details about how action groups and restorative practice might erode boundaries via the enactment of shared roles and the sharing of experiences generating a sense of empathy and collegiality. Our analysis suggests that action groups could engender a new role of a community member, hence building student commitment to a school’s instructional and regulatory orders, in a way that, for many less academic students, classroom interactions probably could not. Our results also confirm our assumption that such erosion would only occur in contexts where there were supportive staff cultures and enabling management structures. However, our results provide the additional insights that a key enabler of action groups eroding boundaries was sufficient numbers of staff on action groups to enable sharing and that action groups achieve broader impacts through initiating broader student involvement or making decisions that affect students more broadly.

Our analysis also broadly supports the mechanism proposed in the intervention theory of change for eroding boundaries among students, providing the additional insight that this occurs via providing students with the social and emotional skills as well as the empathy to develop more caring relationships. Our analysis suggests that such mechanisms were contingent on context: restorative practice only contributed to such mechanisms when this was perceived as necessary and where supporting cultures and structures enabled its delivery, and the classroom curriculum only contributed where it was delivered with fidelity to students in need of it. In contrast, our results provided little evidence of the intervention eroding boundaries between academic learning and students’ broader development. There was no evidence that the intervention achieved a fundamental transformation of schools’ instructional order.

Moreover, our findings suggest that legitimating student expertise and enabling student agency was critical to both the action group and restorative practice [24]. Whereas normal classroom interactions and punitive disciplinary processes cast the student in an essentially passive role, with a body of educational literature portraying student agency in subversive or corrosive terms [44], the action group and restorative sessions gave students more leeway in expressing their perspectives and contributing to solving problems, giving students the role of active community member and not a merely passive learner. Such roles could be said to go some way towards reconstructing students as communitarian participants rather than merely utilitarian clients within the school.

Now, let us turn to drawing on our findings to propose realist-informed CMO configurations. Our findings are consistent with a realist approach to understanding how interventions work. Our results present interventions as merely introducing resources into a setting. Any outcomes arise not directly from these resources but as a result of their being used by local actors, which can then trigger mechanisms which may or may not interact with context to generate outcomes. Our findings present examples where processes started and stalled due to the agency of students or staff or the structural features of the school environment. Our findings suggest it is the mechanisms which are important rather than the specifics of the intervention resources. In some schools, where these particular resources may not be available, our focus on mechanisms may indicate how other resources could activate similar mechanisms in similar contexts [45]. For example, in schools where some staff have additional capacity and recognise the need for better student/staff relationships and where holding an action group is not feasible, students and staff could share experiences, improve bonds, and create new roles via interactions through extra-curricular clubs or by having more staff on already existing student councils.

Our findings enable us to define three CMO configurations, informed by realist evaluation literature [32, 46]. The first CMO configuration relates to increasing students’ commitment to the school. In schools that possess both the management capacity to run action groups successfully and a supportive pre-existing ethos of wishing to involve students in decision-making (context), students will increase their commitment to school via sharing of experiences and perspectives between staff and students in a safe space, improving staff-student relationships, creation of new roles of community members for both students and staff, and transformation of broader student attitudes (mechanisms), and will decrease bullying (outcome). These mechanisms will only remain active when students can see that they are being listened to by teachers and making a tangible difference to the school.

The second CMO configuration relates to the development of pro-social skills and their application to interpersonal conflict. In schools where a critical mass of staff identify improving students social and emotional skills is necessary and they are willing to deliver the curriculum and/or restorative practice approaches to behaviour management (context), formal teaching of such skills in classrooms or in restorative practice sessions as well as by informally modelling such skills in their everyday interactions with students will increase students’ ability to use pro-social skills to address interpersonal conflict (mechanisms) and consequently decrease bullying and aggression (outcome).

The third CMO configuration related to the use of a restorative practice to de-escalate instances of conflict and reduce perpetration. When sufficient staff are willing and trained to deliver restorative practice in a way that enables students to feel safe sharing their experiences and considering the consequences of their behaviour (context), providing conflicting parties with the chance to share perspectives, learn empathy, take responsibility for their actions, and reintegrate themselves into the school community, they will defuse conflict (mechanisms) and subsequently reduce bullying and aggression (outcome).

We will explore the plausibility of these CMO configurations further, but we believe that these configurations could help inform better design of whole-school interventions to address these mechanisms. Realist approaches more generally offer a more nuanced approach to the design of interventions across settings by being clearer about the distinctions between intervention resources and the mechanisms these can trigger when used, as well as by theorising how mechanisms are contextually contingent and outcomes are emergent [45]. In the case of whole-school interventions, this suggests the need for intervention development and evaluation to focus on initiating contextually relevant means of building student commitment, modelling healthy relationships and teaching pro-social skills, and de-escalating conflict.

## Data Availability

The data analysed in this study are not available due to challenges in removing all identifiable information and descriptions.
