# Correction to: Using qualitative research to explore intervention mechanisms: findings from the trial of the learning together whole-school health intervention

**DOI:** 10.1186/s13063-020-04753-w

**Published:** 2020-09-23

**Authors:** Emily Warren, G. J. Melendez-Torres, Russell Viner, Chris Bonell

**Affiliations:** 1 Faculty of Public Health and Policy, London School of Hygiene & Tropical Medicine, London, UK; 2 University of Exeter, Exeter, UK; 3 UCL Institute of Child Health, London, WC1N 1EH UK

**Correction to: Trials (2020) 21:774**

**https://doi.org/10.1186/s13063-020-04688-2**

Following publication of the original article [1], we were notified of a mistake in the spelling of the second author.

- Incorrect spelling: Meledez-Torres
- Correct spelling: Melendez-Torres

The original article has been corrected.
